# Radiolucent lines in low-contact-stress mobile-bearing total knee arthroplasty: a blinded and matched case control study

**DOI:** 10.1186/1471-2474-12-142

**Published:** 2011-06-29

**Authors:** Patrick Sadoghi, Andreas Leithner, Patrick Weber, Jörg Friesenbichler, Gerald Gruber, Norbert Kastner, Katrin Pohlmann, Volkmar Jansson, Bernd Wegener

**Affiliations:** 1 Department of Orthopaedics, Ludwig-Maximilians-University Munich, Campus Grosshadern, Marchioninistrasse 15, 81377 Munich, Germany; 2 Department of Orthopaedic Surgery, Medical University of Graz, Auenbruggerplatz 5, 8036 Graz, Austria; 3 Altona Children' s Hospital, Bleickenallee 38, 22763 Hamburg, Germany

**Keywords:** Arthroplasty, low-contact-stress, mobile-bearing, radiolucent lines, knee pain

## Abstract

**Background:**

Low-contact-stress (LCS) mobile-bearing total knee arthroplasty (TKA) (Johnson & Johnson, New Brunswick, NJ; previously: DePuy, Warsawa, USA) provides excellent functional results and wear rates in long-term follow-up analyses. Radiological analysis shows radiolucent lines (RLL) appearing immediately or two years after primary implantation, indicative of poor seat. Investigations proved RLL to be more frequent in uncemented TKA, resulting in a consensus to cement the tibial plateau, but their association with clinical findings and patients discomfort and knee pain is still unknown.

**Methods:**

553 patients with 566 low-contact-stress (LCS) total knee prostheses were screened for continuous moderate knee pain. We compared tibial stress shielding classified by Ewald in patients suffering from pain with a matched, pain-free control group on blinded X-rays. We hypothesized a positive correlation between pain and radiolucency and higher frequency of such radiolucent lines in the most medial and most lateral zones of the tibial plateau.

**Results:**

Twenty-eight patients suffered from knee pain in total. Radiolucencies were detected in 27 of these cases and in six out of 28 matched controls without knee pain. We could demonstrate a significant correlation of knee pain and radiolucencies, which appeared significantly more frequently in the outermost zones of the tibial plateau.

**Conclusion:**

Our findings suggest that radiolucent lines, representing poor implant seat, about the tibial plateau are associated with knee pain in LCS patients. Radiolucencies are observed more often in noncemented LCS, and cementing the tibial plateau might improve implant seat and reduce both radiolucent lines and associated knee pain.

## Background

Low-contact-stress (LCS) mobile-bearing total knee arthroplasty (TKA) (Johnson & Johnson, New Brunswick, NJ; previously: DePuy, Warsawa, USA) provides excellent functional results and wear rates in long-term follow-up analysis [1-4]. The LCS is designed to increase the functional range of motion (ROM) and reduce wear, in comparison to other fixed bearing TKA designs [5]. Radiological analysis reveals radiolucent lines (RLL) appearing immediately or two years after primary implantation [6-8]. RLL are radiolucent intervals between the cement/implant and the adjacent bone caused by imperfect tibial cuts or excessive micromotion, leading to poor implant seat [6,9-15].

Earlier studies demonstrated RLL to be more frequent in uncemented TKA, resulting in a consensus to cement the tibial plateau [16,17], yet the potential and likely association between clinical findings and radiolucencies was never formally assessed [10,11,18,19].

Therefore, this multicenter study screened 553 patients with 566 low-contact-stress total knee prostheses for continual moderate knee pain determined by the Knee Society Score [20]. Selected patients were further tested for tibial stress shielding, evaluated by the classification of Ewald et al. [21] and compared to a pain-free, age- and sex-matched control group selected from the same population of 566 patients.

The primary purpose of this study was to test for a correlation between knee pain and tibial radiolucent lines. The secondary purpose was to describe the distribution of radiolucencies around the tibial plateau.

The study hypothesis was, that knee pain correlates with tibial radiolucent lines and that these radiolucent lines appear most frequently in the most lateral and most medial zones of the tibial plateau than in more central areas.

## Methods

Due to the retrospective nature of this study using anonymous data, after contacting the ethics committee it was stated, that ethical approval was not necessary at both universities.

### Study Design and Patient Recruitment

This study was designed as an age and sex matched case control study. A sample of 553 anonymous patients with 566 LCS implants was reviewed. The indication for knee replacement was based on subjective pain level, a continual need for analgesic and anti-inflammatory drugs, and objective functional limitations such as reduced walking distance and decreased range of motion. Osteoarthritis of the knee joint was verified by use of anterior-posterior and lateral X-rays. All procedures were done between January 1981 to June 2003 at two different institutions by five different surgeons.

From this sample we excluded patients with septic tibial loosening, low-grade infections, or aseptic tibial loosening (n = 13) evaluated by detailed laboratory screening (CRP, leukocytes) or scintigraphy or any kind of femoral loosening (n = 24) as the cause (septic loosening and low-grade infections evaluated by laboratory results, aseptic loosening without signs of infections). In addition, we excluded all patients with aseptic tibial or femoral loosening, which was verified using scintigraphy (n = 43). Furthermore we excluded patients with polyethylene wear, a postoperative antero-posterior long-leg standing X-ray axis of more than 5 degrees of varus or valgus, any soft tissue impingement, patellar complications, infections, reflex sympathetic dystrophy, flexion contracture, inadequate flexion, (radiological or intraoperative) signs of notching, overhang, overstuffing or undercutting and clinical instability (n = 67). From the remainder we included patients with at least "moderate" knee pain on the KSS were as cases, and an equal number of age- and sex-matched, pain-free patients as controls.

Anonymized clinical and radiological data for all patients were obtained from a database of digitalized patient records. All patients included in this database gave informed consent to participate in the follow-up and were aware, that anonymized, aggregate data will be used for research. Therefore after contacting the ethics committee it was stated, that approval by the ethic committee was not necessary due to the retrospective nature using anonymous data.

### Surgical technique and rehabilitation

Procedures were done in general or epidural anaesthesia with a medial parapatellar or transquadricipital approach in a consecutive series at each institute. Five orthopaedic surgeons operated on the patients in total. All surgeries were performed by or under direct supervision of the head of the division of arthroplasty of both institutes. First the tibial osteotomies were performed, according to the LCS guidelines from DePuy with a posterior slope of 5° using an extramedullary system. The femoral cut was performed with an intramedullary guide system providing 3° of external rotation using the posterior condyles as reference points. Patella resurfacing was not done. This technique was applied using totally cemented (tibial and femoral) and only tibial cemented prosthesis according to the patient age and bone quality. Tibial and femoral cementation was performed with standardized methods according to the manufacturer' s instructions using Refobacin Bone Cement R (RBC) (AAP Biomaterials GmbH & Co. KG, Dieburg, Germany;) and Palacos R + G (PRG) (Heraeus Medical GmbH, Hanau, Germany). Constituents were stored at room temperature (23°C) before preparation. The samples were mixed for 30 seconds after the addition of all the powder to monomer, under a vacuum of 200 millibar using the Easymix cement injector (Coripharm GmbH & Co KG, Dieburg, Germany). No fractionation of the tibial plateau and the femoral shield was performed in any case. All knees were closed in layers with two drains in place, which were removed after 48 hours. which was not proven to restore the long-leg standing axis more appropriately [22]. No single implant showed signs of notching, overhang, overstuffing or undercutting in intraoperative examination or on postoperative X-rays.

Postoperatively, patients were allowed full weight bearing and continuous passive motion was used as of the second postoperative day. All patients were discharge between 10 and 14 days postoperatively and referred to an outpatient rehabilitation program until their six week follow-up.

All patients in both clinics had standardized pain management protocols: Neodolpasse i.v. (combining 75 mg diclophenac and 30 mg orphenadine) was given twice a day and Pantoloc p.o. (pantoprazol 40 mg) once a day for at least ten days. Patients with allergies or kidney disorders were given Novalgin i.v. (metamizol 1 g) three times a day in combination with Pantoloc p.o. (pantoprazol 40 mg) once. In case of further pain, an injection of Dipidolor i.m. (piritramide 7.5 mg) was given every 4 hours.

### Outcome assessment

At 2 to 10 year follow-up the patients were clinically evaluated using the WOMAC score and Knee Society Score [20]. In case of "continual moderate pain or more" on the KSS score, the patients' X-rays at minimum 2-year follow-up were re-evaluated. Two blinded observers (P.S. and J.F.) re-evaluated the anonymous anterior-posterior X-rays of all included patients independently, assessing location and intensity of radiolucent lines according to Ewald et al. [21]. All detected "zones" out of four measurements by two observers in patients with/without knee pain were summarized and we calculated the actual relative number of a possibly affected number of a sum of all four measurements. A potential limiting factor for this assessment was the upper limit of magnification of 72 pixels per inch, corresponding to 804 pixels times 963 pixels, which is a technological constraint of the picture viewing software.

### Statistical analyses

Independent t-tests were used to compare demographic parameters across groups. The observer agreement of the radiological evaluation was evaluated using the Cohen's kappa coefficient. The correlation between the pain ("continual moderate pain" or worse in the KSS) and radiolucent lines was evaluated using Pearson's coefficient. We evaluated post hoc power according to the method by Hoening and Heisey [23] All calculations were done using SPSS 13.0 (SPSS Inc., Chicago, IL). A *P*-value of less than 0.05 was considered to be significant.

## Results

### Demographic Data

The patients' mean age (including 533 patients) at time of surgery was 68.3 years (range: 39 to 89). The mean age at mean clinical follow-up of 7.3 years (range: 2 to 10) was 75.6 years (range: 44 to 95). Demographic data is presented in table 1. We included 388 female (72.8%) and 145 male (23.2%) patients in this analysis and 44.8% of the prosthesis were implanted in right knees with 55.2% in left knees. 271 LCS prostheses were totally cemented (tibial and femoral) and 295 prostheses with a cemented tibial plateau only. No revision surgery was performed in any of the reported 56 cases. Implant sizes are given in table 2.

**Table 1:** Twenty-eight out of 566 low contact stress (LCS) total knee arthroplasties (TKA) in 533 patients who suffered from moderate knee pain were matched to 28 patients not suffering from any pain in terms of age, sex and radiological follow-up

	patients with continual moderate knee pain, n = 28	matched patients without any knee pain, n = 28	*P*- value
age in years*	68.3, 49-89	68.8, 43-86	> 0.82

sex ratio m:f	1: 2.68	1: 2.72	> 0.62

radiological follow-up in months*	22.2, 18-24	22.4, 19-24	> 0.59

Note that statistical analysis revealed no significant difference in demographic data between both groups.

* numbers are reported in mean, range.

**Table 2:** Twenty-eight out of 566 low contact stress (LCS) total knee arthroplasties (TKA) in 533 patients who suffered from moderate knee pain were matched to 28 pain-free patients in terms of age, sex and radiological follow-up

component size	patients with knee pain in mobile bearing LCS TKA, n = 28	matched patients without knee pain in mobile bearing LCS TKA, n = 28
		

Femoral shield		

Large plus	3	3

Large	5	6

Standard plus	8	8

Standard	7	7

Medium	2	2

Small medium	1	0

Small	2	2

		

Tibial plateau		

7	0	0

6	0	0

5	0	0

4	0	0

3	15	16

2.5	11	10

2	2	2

		

Inlay size		

15 mm	3	3

12.5 mm	17	16

10 mm	9	8

The sizes of implanted components of the LCS system (DePuy, Warsawa, IN) are listed below for both groups.

### Clinical Results

At follow-up (range 5 to 10 years) the mean active range of motion (ROM) of all 566 prostheses was 102.4 degrees (range: 5 to 145). The mean WOMAC score [20] (was 27.48 points (range: 0 to 84). The mean KSS for pain [20] was 82.95 points (range: 0 to 100) and the mean KSS for function [20] was 67.34 points (range: 0 to 100). Twenty-eight patients (26 totally cemented and 2 tibial only) suffered from "continual moderate pain or more" as the first question of the KSS score for pain and were re-evaluated for radiolucent lines as previously described, together with 28 randomly chosen, pain-free controls.

### Radiolucencies and Correlation with Pain

**Figure 1:**
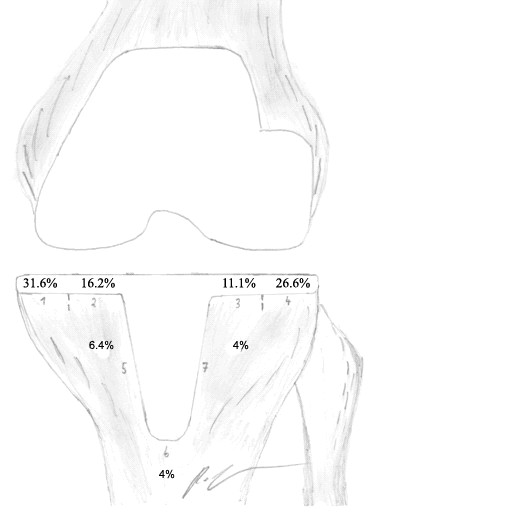
**Figure illustrating the seven possibly affected zones of tibial stress shielding, according to the classification of Ewald et al. **[20]** with the percentage distribution in terms of 28 cases of LCS total knee replacements with continual moderate knee pain**. Twenty-seven cases showed radiolucent lines in total (Table 2).

**Figure 2:**
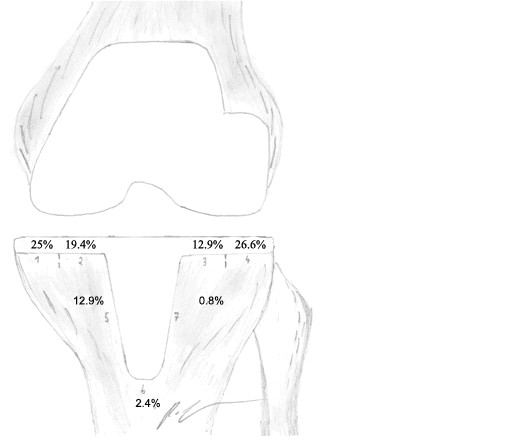
**Figure illustrating the seven possibly affected zones of tibial stress shielding, according to the classification of Ewald et al. **[20]** with the percentage distribution in terms of 31 age, sex and radiological follow-up matched cases of LCS total knee replacements without any knee pain**. Nine cases showed radiolucent lines in total (Table 2).

Radiolucent lines were detected in 27 out of 28 patients with pain and in six out of 28 matched pain-free patients (Table 3). The relative distribution of affected tibial zones in pain-free patients and patients with continual moderate knee pain is shown in Figure 1, 2, and 3. Interobserver aggreement was high with a kappa coefficient of 0.781.

**Table 3:** Distribution of differently affected tibial zones of stress shielding, according to Ewald [21] after implantation of a low contact stress (LCS) total knee arthroplasty (TKA)

	Relative numbers of affected „zones" of tibial stress shielding							
	Zone "1"	"2"	"3"	"4"	"5"	"6"	"7"	Sum

								

RLL in patients with knee pain, n = 27 (Figure 1)	31.6%*	16.2%	11.1%	26.6%	6.4%	4%	4%	100

RLL in matched patients without knee pain, n = 6 (Figure 2)	25%	19.4%	12.9%	26.6%	12.9%	2.4%	0.8%	100

**Figure 3:**
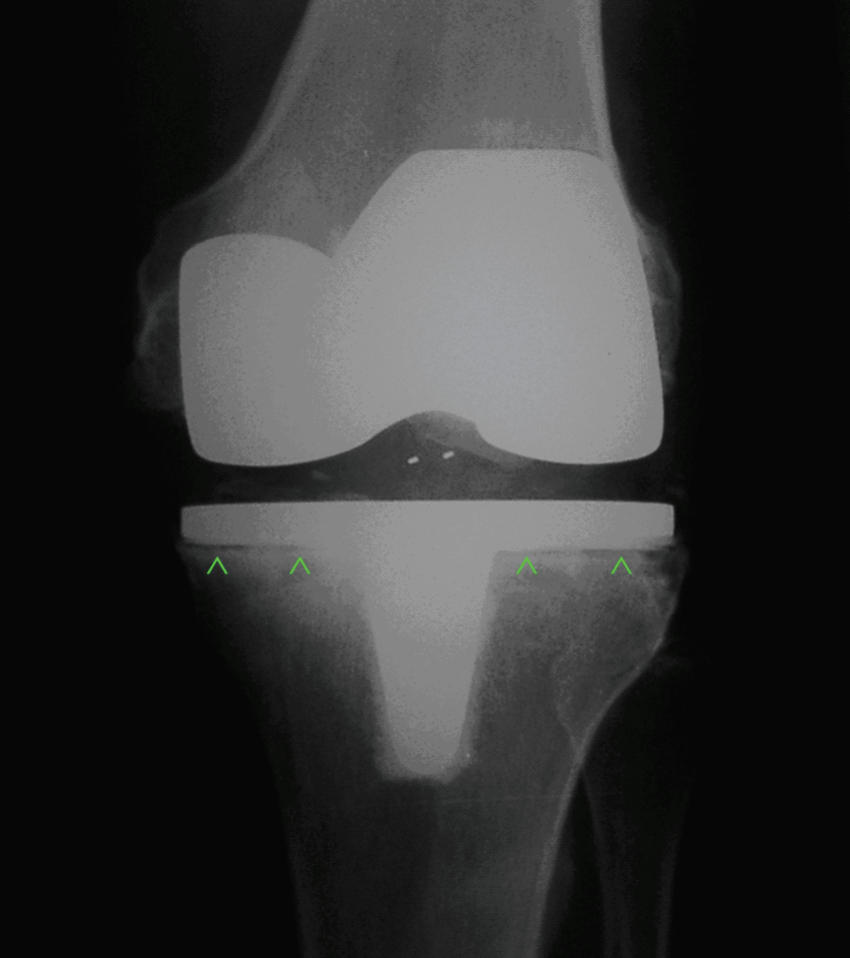
**Anterior-posterior X-ray of a left Low-contact-stress total knee prosthesis (with cemented tibia) in a patient with continuous moderate knee pain with tibial stress shielding in zones "1", "2", "3", and "4", according to the classification of Ewald et al. **[20].

Tibial zones of stress shielding were detected in 27 out 28 patients suffering from continual moderate knee pain (Figure 1, 3) and in 6 out of 28 age, sex, and follow-up matched pain-free patients (Figure 2). We present relative numbers of detected radiolucent lines (RLL) of a sum of four measurements by two observers with a minimum break of two weeks between. Accuracy of our measurements was evaluated with a "good" agreement of a Cohen' s kappa coefficient of p = 0.781.

* These percentages show the actual relative number of a possibly affected number of a sum of four measurements by two observers.

* Figure 3 shows an example of an anterior-posterior X-ray of a left Low-contact-stress total knee prosthesis (with cemented tibia) in a patient with continuous moderate knee pain with tibial stress shielding in zones "1", "2", "3", and "4".

We found a significant correlation of pain with tibial radiolucent lines with a p-value of 0.824 of the Pearson' s coefficient. Furthermore we saw that RLL in patients with continual moderate pain appeared significantly more frequently (p < 0.001) in the most medial/ lateral zones of the medial "zone 1" (31.6%) and lateral plateau "zone 4" (26.6%) than in less medial/lateral zones or the stem zone "zone 2 to 7" (16.16%, 11.1%, 6.4%, 4%, 4%) (Figure 1). No revision surgery due to radiolucencies was performed in any of the reported 56 cases. Post-hoc power according to Hoening and Heisey of this observation was over 80% [23].

## Discussion

The primary purpose of this study was to correlate subjective knee pain with tibial radiolucent lines in patients with cemented LCS total knee replacement. The secondary purpose was to analyze the spatial distribution of these radiolucencies. We observed a strong correlation between knee pain and tibial radiolucent lines, and found that these lines appear more frequently in the most lateral and most medial zones ("zone 1" and "zone 4") of the tibial plateau.

Parsch et al. [4] showed 6.7% of tibial radiolucencies in their patient population without further division among patients suffering from continual moderate knee pain or tibial zones according to Ewald et al. [21]. This is in line with Whitehead et al. [24] demonstrating radiolucent lines in 6% of the tibial components and Vogt et al. [25] with 7% of tibial radiolucencies appearing in cemented (n = 2) and cementless (n = 1) prosthesis. Our data revealed 27 out of 28 cases with pain, respectively 6 out of 28 pain-free cases of tibial radiolucencies facing the fact, that the authors only evaluated 28 patients with pain and 28 matched pain-free patients out of 566 LCS TKA in total. If detected, these radiolucent lines appeared more frequently in the most medial/lateral zone of the tibial plateau in both groups, patients with and without pain.

According to Aebli et al. [26] radiolucent lines might occur due to imperfect cuts of the tibial plateau or due to micromotions leading to the formation of gaps, which may prevent osteointegration in uncemented TKAs inducing the formation of fibrous tissue or regions of osteoporosis. This is in line with Toksvig-Larsen and Ryd [27] reporting a 1 to 2 mm gap between the lowermost and uppermost points of the tibial plateau after cutting, which might result in tibial stress shielding. This, in turn, would be a plausible reason for moderate knee pain.

In addition, the size of the tibial anchoring pad is larger than the femoral, making it more difficult to achieve adequate press-fit to avoid tibial stress shielding as another cause for patients discomfort after implantation of a LCS TKA [27].

In order to guarantee adequate measurements and detection of tibial radiolucent lines, the authors performed an inter- and intraobserver correlation analysis of two observers resulting in a "good" agreement. However, according to Vyskocil et al. [28] radiolucent lines with a width less than 2 mm may not appear on conventional anterior-posterior radiographs if the central stem is tilted about 2.3° to a tibial component with a width of about 50.0 mm. Thereafter, radiolucent lines should be divided in zones and labelled as either "present" or "absent" as performed in this study [28].

### Limitations and benefits

This study could demonstrate a significant correlation in patients with continual moderate knee pain with radiolucent lines, which predominantly appear in the most medial and most lateral zones of the tibial stem, but we lack further radiological information of the occurrence of possible radiolucencies in the remaining 510 pain-free patients or patients reporting only very discrete knee pain. Furthermore, we only evaluated standard anterior-posterior radiographs without data of fluoroscopically-assisted radiographs. Next, surgeries were not performed by one single surgeon but by five different surgeons in total, which could have possibly leaded to further bias of the data. However, implantation of total knee arthroplasty is a procedure only open to experienced surgeons in both clinics and mostly performed under supervision of the head of the division of arthroplasty. In addition, patients of this study had relatively long hospitalization time (average of 2 weeks), which is typical for the authors' countries but might differ from other European or US countries. Furthermore, we did not correlate the initial time when pain was reported with the first appearance of radiolucent lines but those two parameters (pain and RLL) at follow-up in general.

It should be noted, that with the sample size of 56, the magnitude of differences in affected tibial zones with radiolucencies was large enough that post hoc power analysis revealed over 80%. Last, we are able to present the radiological and clinical data from all patients who suffered from continual moderate knee pain matching our inclusion criteria.

## Conclusion

We could demonstrate a significant correlation of continual moderate knee pain and the appearance of tibial radiolucent lines and that these radiolucent lines appeared significantly more frequently in most medial and most lateral zones. We believe that in case of no further specified pain after implantation of the LCS prosthesis, radiolucencies, which do not imply implant loosening, should still be suspected as the possible cause of pain. Surgeons should try to avoid the appearance of radiolucencies by implanting cemented tibial plateaus using perfect cuts and avoiding uncemented TKA systems.

## Competing interests

There exist no financial or non-financial competing interests in case of any author of this manuscript. No benefits or funds were received in support for the study.

## Authors' contributions

PS: preparation of the manuscript, data collection, study design; AL: revision of the manuscript, statistical advice; PW & JF: revision of the manuscript, statistical analysis, data analysis; GG & NK: revision of the manuscript; operating surgeons; KP: data collection, study design; VJ: revision of the manuscript, operating surgeon; BW: study design, revision of the manuscript. All authors read and approved the manuscript.

## Pre-publication history

The pre-publication history for this paper can be accessed here:

http://www.biomedcentral.com/1471-2474/12/142/prepub
